# Draft genome sequence of multidrug-resistant *Escherichia coli* isolated from retail chicken liver in Kosovo

**DOI:** 10.1128/mra.01033-25

**Published:** 2026-02-18

**Authors:** Anahita Ghorbani Tajani, Afrim Hamidi, Aniket Sharma, Driton Sylejmani, Erënesa Gorçaj, Gente Hashani, Bardhyl Noci, Bledar Bisha

**Affiliations:** 1 Department of Animal Science, University of Wyoming 4416, Laramie, Wyoming, USA; 2 Faculty of Agriculture and Veterinary, University of Pristina, Pristina, Kosovo; University of Maryland Baltimore, Baltimore, Maryland, USA

**Keywords:** *Escherichia coli*, antimicrobial resistance, Kosovo, public health surveillance

## Abstract

We report the draft genome of multidrug-resistant *Escherichia coli* UA1 from Kosovo retail chicken liver, revealing ARGs (*bla*_EC-18_, *bla*_CTX-M-55_, and *dfrA36*), virulence genes, and plasmids. Assembled into 86 contigs and 214 scaffolds, it highlights antimicrobial resistance dissemination in the food chain.

## ANNOUNCEMENT

Antimicrobial resistance (AMR) in foodborne bacteria is a growing public health concern, requiring surveillance in understudied regions to guide mitigation (1). We report the draft genome of multidrug-resistant *Escherichia coli* from Kosovo retail chicken livers, characterizing its genomic content, resistance genes, plasmids, and pathogenicity potential.

The isolate was obtained from retail chicken livers in Kosovo (retail acquisition; no human/animal subjects). According to the NARMS protocols (2), approximately 50 g chicken liver was rinsed in 250 mL BPW by manual massaging for 3 min; a 50 mL rinsate aliquot was enriched in 50 mL double-strength MacConkey broth (35°C, 24 h); streaked onto MacConkey agar (35°C, 24 h); and a typical colony was subcultured on blood agar (35°C, 18–24 h). *E. coli* was confirmed by MALDI-TOF MS (Bruker Microflex LRF, Biotyper v3). MIC assays were performed according to the CLSI M100-Ed35 guidelines (3) using CMV3GANF plates, with incubation at 37°C (16–20 h) and analysis using SWIN v3.3 software. Resistance was observed to azithromycin, cefoxitin, ceftriaxone, ciprofloxacin, gentamicin, streptomycin, sulfisoxazole, and tetracycline; intermediate susceptibility was observed to ceftiofur.

For genomic DNA, a single colony was subcultured overnight (18 h) in brain heart infusion broth at 37°C, and DNA was extracted using the DNeasy PowerLyzer Microbial Kit (Qiagen). DNA quantity/quality was assessed using a Qubit fluorometer. Library preparation used the Nextera XT DNA Library Prep Kit (Illumina) following the manufacturer’s workflow; genomic DNA was sonicated to ~350 bp, end-repaired, A-tailed, adaptor-ligated, and PCR-amplified. Sequencing was performed by CD Genomics on an Illumina NovaSeq 6000 platform, generating 233 bp paired-end reads, yielding 9,019,931 read pairs (~823× mean coverage). Read quality was assessed using FastQC v0.12.1 (4), and the reads were trimmed using Trimmomatic v0.39 (5). Assembly was performed using SPAdes v3.15.5 (6), and annotation using the NCBI Prokaryotic Genome Annotation Pipeline (PGAP; v6.8) (7). ARGs were screened using ABRicate v0.2-0 (8), plasmid replicons using PlasmidFinder v2.0.1 (9), serotype using SerotypeFinder v2.0 (10), MLST using mlst v2.23.0 (11), virulence genes against the VFDB using VirulenceFinder v2.0 (12), fimH allele assignment using FimTyper v1.0 (13), and pathogenicity probability using PathogenFinder v2.0 (14). Default parameters were used for all software unless otherwise noted.

The draft assembly is 5,192,718 bp with 51% GC content, assembled into 86 contigs (contig N_50_ ≈ 150 kb) and 214 scaffolds (scaffold N_50_ = 357,836 bp). Annotation predicts 5,088 genes. ARGs detected include *bla*_EC-18_, *bla*_CTX-M-55_, and *dfrA36*; virulence factors include *anr*, *csgA*, *fdeC*, *fimH* (*fimH31*), *gad*, *hlyE*, *lpfA*, *nlpI*, *papA_F19*, *shiA*, *shiB*, *terC*, *tia*, *traT*, *yehA–D*, and *yghJ*. *In silico* typing assigned serotype O8:H7 and ST1642. Plasmid replicon analysis identified four plasmid incompatibility types in the isolate, including IncFIA (AP001918), IncFIB (AP001918), IncQ1 (M28829), and p0111 (AP010962). The machine-learning pathogenicity predictor estimated a human-pathogen probability of 0.937. These genomic findings align with the observed phenotypic resistance and indicate the presence of multiple mobile elements likely contributing to AMR dissemination in the food chain.

This genome aids regional AMR surveillance, comparative studies of foodborne *E. coli* in understudied regions, and ongoing monitoring of retail meat as a source of clinically relevant resistance determinants.

## Data Availability

This Whole Genome Shotgun project has been deposited in GenBank under the accession no. JBPPCA000000000. The version described in this paper is the first version, JBPPCA010000000. Raw reads are available in the NCBI Sequence Read Archive under the accession no. SRR34954221.
